# Correlations Between Social Support and Loneliness, Self-Esteem, and Resilience Among Left-Behind Children in Mainland China: A Meta-Analysis

**DOI:** 10.3389/fpsyt.2022.874905

**Published:** 2022-04-27

**Authors:** Haitao Huang, Xiao Wan, Yipei Liang, Yiming Zhang, Qianwen Peng, Yueming Ding, Guangli Lu, Chaoran Chen

**Affiliations:** ^1^Institute of Nursing and Health, School of Nursing and Health, Henan University, Kaifeng, China; ^2^Institute of Business Administration, School of Business, Henan University, Kaifeng, China

**Keywords:** social support, loneliness, resilience, left-behind children, China, meta-analysis

## Abstract

**Background:**

Social support is frequently reported to be correlated with loneliness, self-esteem, and resilience among left-behind children in mainland China. However, to date, there is no consensus on the extent to which those factors are correlated with social support among left-behind children. We thus performed a meta-analysis to quantitatively synthesize the previous findings.

**Methods:**

Two investigators systematically and independently searched PubMed, EMBASE, Web of Science, PsycINFO, Wan Fang, Chinese National Knowledge Infrastructure (CNKI) and China Science Technology Journal Database (VIP) on January 9, 2022. Pooled Pearson's correlation coefficients between social support and loneliness, self-esteem, and resilience were calculated by Stata 16.0 software using random effects model.

**Results:**

Forty-seven studies involving a total of 30 212 left-behind children were identified. A large degree of negative correlation was found between social support and loneliness [summary r: −0.36 (95% CI: −0.42– −0.30), *p* < 0.001]. Large positive correlations were found between social support and self-esteem, and resilience [self-esteem: summary r: 0.33 (95% CI: 0.24–0.41), *p* < 0.001; resilience: summary r: 0.45 (95% CI: 0.38–0.50), *p* < 0.001]. The pooled correlations revealed some discrepancies when stratified by some moderators. Sensitivity analysis also revealed the robustness of the findings. The Egger regression and Duvall and Tweedle trim-and-fill procedure suggest the absence of publication bias.

**Conclusion:**

The current meta-analysis provided solid evidence that social support has a high degree of negative correlation with loneliness and a high degree of positive correlation with self-esteem and resilience among left-behind children in mainland China. This indicated that left-behind children with high levels of social support tend to have lower levels of loneliness and higher levels of self-esteem and resilience. More studies, especially large prospective studies, are warranted to verify our findings.

## Introduction

Since China's economic reform and opening up in the 1980's, with the rapid economic development and industrialization, a growing number of rural middle-aged and young labor force have migrated to work in the country's main cities in order to improve their family's economic conditions. China has experienced the largest internal migration in human history (1). Due to the high cost of living and education in cities, as well as the limitations of China's dual urban-rural household registration system, many migrant workers are forced to keep their children in the household registration area and entrust their care to family members (usually grandparents), resulting in the formation of a unique group of children known as “left behind children” (LBC). LBC refers to children under the age of 18 who have to stay in their hometown due to one or both parents going out to work, and are supervised by one parent and/or grandparents, relatives, neighbors for more than 6 months (2). In 2018, the Ministry of Civil Affairs of China conducted a survey on left-behind children, which revealed that China's total number of left-behind children had reached a staggering 6.97 million (3). The situation of LBC in China is a major social and health issue that has attracted widespread concern. Many studies show that LBC has more behavioral and psychological problems than non-left-behind children (NLBC) (4–7). Therefore, the study of this group is not only related to their own destiny, but also provides a Chinese perspective for the development of disadvantaged children around the world.

Social support refers to the respect, care and help that individual perceive from social relations around them (such as their family, friends, important others.), which can make individuals avoid or less affected by negative events of stress (8). For the LBC in the family environment with relatively lack of family affection, social support is their coping resources to adapt to the adverse environment (9). Social support has dual effects, namely main effect and buffer effect (10, 11). The first is to enhance the subjective self-evaluation of the individual and mobilize the positive qualities of the individual to improve the ability to adapt to adverse environments, such as improve self-esteem and resilience (12); the second is to directly buffer the impact of stress on the individual and play a protective role in maintaining individual mental health (10).

An increasing amount of evidence has shown that social support is closely related to many detrimental psychological problems of LBC such as loneliness, low levels of self-esteem, and low levels of resilience (13–15). However, there has been no consensus on the extent to which these factors are correlated with social support among LBC so far in mainland China. Specifically, first of all, as for the correlation between social support and loneliness among LBC in mainland China, some studies have found a relatively large negative correlation (14, 16), while others have found a small negative correlation (17, 18). Secondly, regarding the relationship between social support and self-esteem, some studies have found that there was a small positive correlation,while others have found a lager positive correlation (15, 19). Similarly, the strength of identified associations between social support and resilience among LBC in mainland China has varied considerably thus far, ranging from small (r = 0.22) (20) to large (r = 0.75) (21).

Using meta-analysis, the outcomes of several studies can be statistically combined to obtain an overall effect size. However, up to now, no meta-analysis has been conducted on the relationship between social support and loneliness, self-esteem, and resilience among LBC in mainland China. Therefore, the purpose of this study is to conducted 3 meta-analysis to explore the relationship between social support and loneliness, self-esteem, and resilience among LBC in mainland China. The potential existence of publication bias was addressed. Because journals may tend to publish studies with significant results and reject those with non-significant results, this may lead to publication bias; In addition, existing null results that have never been published may lead to overestimation of the relationship between variables (22). Furthermore, subgroup analysis was used to analyze the moderating effects of sampling strategy, sample size, gender, educational stage of children, published type, social support measurement instruments, and the measurement instruments for the three targeted variables in the included studies to evaluate whether the relationships between variables were moderated by demographics and study characteristics.

## Materials and Methods

A systematic review and meta-analysis was conducted according to the Preferred Reporting Items for Systematic Reviews and Meta-Analyses (PRISMA) (23). Moreover, the review was registered in PROSPERO (registration number: CRD42022304140).

### Searching Strategy

The databases PubMed, EMBASE, Web of Science, PsycINFO, Wan Fang database, Chinese National Knowledge Infrastructure (CNKI) and China Science Technology Journal Database (VIP) were searched on January 9, 2022 using the key words “left-behind,” “stay at home,” “child^*^,” “adolescent^*^” “child^*^,” “student^*^,” “support,” “social support,” “social network,” “social relation,” “social resource,” “social environment,” “Chinese” and “China.” Finally, appropriate Boolean operators are used to combine these search terms. In addition, Google Scholar and CNKI were used to conduct gray literature search for dissertations that met our inclusion criteria. A detailed search strategy is available in Supplementary File 1. Publication languages were limited to English and Chinese. Reference lists of retrieved studies were scanned for further possible articles.

### Selection Criteria

Two investigators independently screened the retrieved literature according to the following inclusion and exclusion criteria: (1) LBC living in China; (2) cross-sectional studies offering Pearson's correlation coefficients for the associations between social support and loneliness, self-esteem, and resilience; (3) social support measurement instruments were limited to the Social Support Rating Scale (SSRS), Social Support Questionnaire (SSQ), Multi-dimensional Scale of Perceived Social Support (MSPSS) and Chinese version Perceived Social Support Scale (PSSS); (4) loneliness measurement instruments were limited to Children Loneliness Scale (CLS), University of California Los Angeles Loneliness Scale (ULS), Mental Health Diagnostic Test (MHT) and Adolescents' loneliness scale (ALS); (5) self-esteem measurement instruments were limited to Self-Esteem Scale (SES) and Collective Self-esteem Scale (CSES); (6) there was no restriction on resilience scales; and (7) published in English or Chinese; The exclusion criteria are as follows: (1) “special” left-behind children, such as orphans or children from single-parent families; (2) conference abstracts and review articles. If more than one paper were published based on the same dataset, we only included the articles using more completed information; and (3) literature with poor quality or apparent data mistakes were also excluded.

### Study Selection and Data Extraction

Two researchers (HTH and XW) systematically and independently evaluated the eligibility of the study and extracted data. In case of disagreement during the process, it will be resolved through discussion or consultation with a third researcher (YMD). The following information was extracted: first author, year of publication, geographical area, sampling strategy, sample size, number of males and females, mean age, measurement tool of social support level, instruments used to measure levels of loneliness, self-esteem, and resilience and Pearson's correlation coefficients between social support and the above three variables.

### Assessment of the Study Quality

Nine-item Joanna Briggs Institution Critical Appraisal Checklist for Studies Reporting Prevalence Data is used as a quality assessment tool (24). A minor adjustment has been made to the third item of the scale. That is, the appropriate sample size was judged based on Pearson's correlation study design rather than the prevalence study design. The answers to each item include “yes,” “no,” “unclear” and “not applicable.” If the answer is “yes,” the item will receive one point; otherwise, it will receive zero points. The higher the score, the better the quality of the method. The methodological quality of all studies included was independently assessed by two researchers (HTH and YMD). A third author was available for resolving differences (CRC). The results showed that all the included studies were of medium or high quality (total score ≥ 6). See Supplementary File 2 for the specific quality evaluation results.

### Statistical Analysis

Stata 16.0 was used for statistical analysis (STATA Corp, College Station, TX). The Pearson product-moment correlation coefficient (r) is used as the effect size of this study. Since the variance is strongly dependent on the correlation, the r coefficient is transformed by the formula into Fisher 'z (25). The sample correlation r is converted into Fisher's Z by formula (1), and the standard error is calculated by formula (2), where n is the sample size. Fisher z statistics are assumed to be normally distributed data, and their 95% confidence intervals are calculated by formula (3). In the end, an inverse transformation was performed to report the results on the scale of the r-coefficient by formula (4). Moreover, since the reference standard for the interpretation of the correlation coefficient suggested by Cohen (26) (r = 0.1 is low correlation, r = 0.3 is moderate correlation and r = 0.5 is strong correlation) is based on qualitative analysis, which is relatively subjective, so this study adopts the suggestions of Gignac and Szodorai, and r = 0.1, r = 0.2 and r = 0.3 represent relatively small, typical, and relatively large correlation (27).

-
$Fisher^{{}^{\prime }}s\;Z=0.5\ln\frac{1+r}{1-r}$

-
$S_{E}=\sqrt{1/\left(n-\;3\right)}$

- 95*%CI* = *Z*± 1.96(*SEz*)
- $Summary\;r=\frac{e^{2z}-1}{e^{2z}+1}$ (Z=summary Fisher′s Z).

The random-effects model was used for data analysis because it does not assume a common potential effect size for all included studies, making it more suitable than fixed-effects models for meta-analyses based on existing literature (22). The meta-analysis was performed with the Der-Simonian and Laird's method (28), where the weighting of sample size was introduced into the model as the inverse of variance. Heterogeneity was tested by Q statistics and I^2^, which measure whether there are differences between the included studies. In addition, we further tested the probable variables that could moderate the correlations between social support and loneliness, self-esteem, and resilience among LBC by subgroup analysis. Studies were grouped by a few available study characteristics, including sampling strategy, sample size, gender, educational stage of children, published type, social support measurement instruments, and the measurement instruments for the three targeted variables. The Q statistic was also used to test the differences between and within groups of studies.

To assess the impact of individual studies on the summary correlation coefficients and evaluate the robustness of the correlations between social support and loneliness, self-esteem, and resilience, sensitivity analyses were conducted by sequentially omitting one study each turn. Lastly, visual inspection of funnel plots, Egger's linear regression test (29) and Duval and Tweedie's trim-and-fill (30) analysis were performed to help us assess publication bias.

## Results

### Study Selection and Study Characteristics

**Figure 1:**
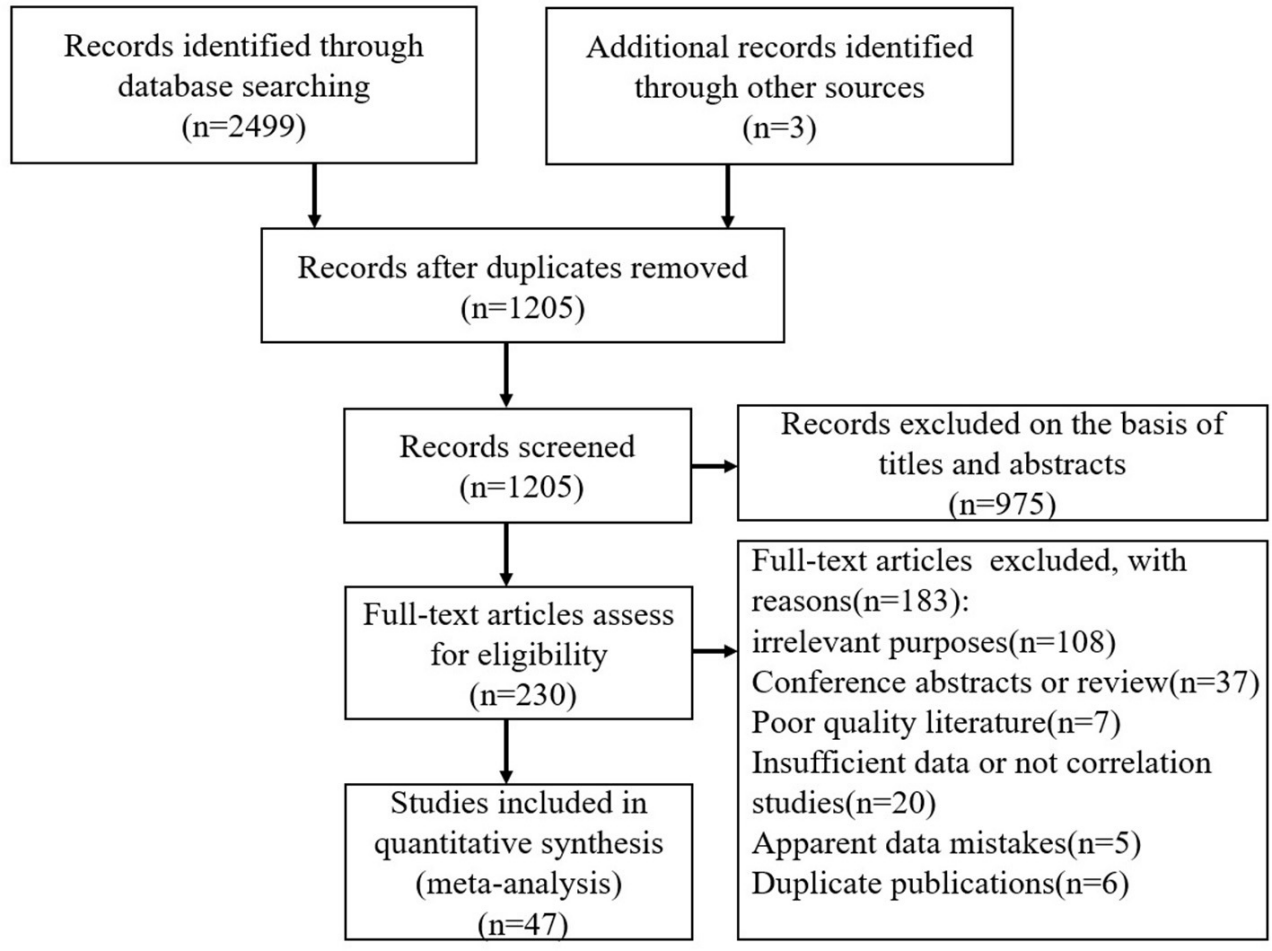
The flow chart of the study selection process.

Our search strategy identified 1 205 studies without duplicates (Figure 1). There were 975 studies excluded after title and abstract screening. Finally, the full texts of 230 articles were reviewed. We excluded 183 studies for the following reasons: irrelevant purposes (*n* = 108), conference abstracts or reviews (*n* = 37), poor quality (*n* = 7), had insufficient data or not correlation studies (*n* = 20), had apparent data mistakes (*n* = 5), or duplicate publications (*n* = 6). As shown in Table 1, 47 studies ultimately met the inclusion criteria, involving a total of 30 212 LBC in China. Of these 47 studies, 20 reported Pearson's correlation coefficients between social support and loneliness, 14 reported Pearson's correlation coefficients between social support and self-esteem, and 20 reported Pearson's correlation coefficients between social support and resilience.

**Table 1:** Characteristics of studies included in the meta-analysis.

**Name (year)**	**Province**	**School year**	**LBC (*n*)**	**Male/** **female**	**Age range/ mean ±SD**	**Sampling method**	**Published types**	**Social support measurement**	**Measurement instrument (Pearson's r)**		
									**Loneliness**	**Self-esteem**	**Resilience**
Liu et al. (31)	Henan	P, J	181	88/93	10–16	cluster sampling	Journal	SSQ	CLS	N/A	N/A
Zhao et al. (32)	Henan	P, J	218	N/A	14.09	convenience sampling	Journal	SSQ	CLS	N/A	N/A
Chen et al. (17)	Heilongjiang	P, J, S	338	N/A	13–15	cluster sampling	Journal	SSRS	MHT	N/A	N/A
Du (33)	Anhui	J	455	214/241	15.58 ± 0.04	random	Journal	SSRS	MHT	N/A	N/A
Li et al. (34)	Zhejiang	J	561	51.5	N/A	convenience sampling	Journal	SSRS	N/A	N/A	ERS
Li (35)	Henan	J	386	52.6	11.62 ± 0.58	random	Journal	SSRS	N/A	N/A	ERS
Liu (36)	Sichuan	J	1,016	49.3	N/A	convenience sampling	Dissertation	PSSS	N/A	N/A	ERS
Wu et al. (13)	Guangdong	P, J	437	N/A	11–16	random	Journal	SSRS	CLS	N/A	N/A
Zeng (18)	Henan	J	506	37	N/A	convenience sampling	Journal	SSRS	MHT	N/A	N/A
Chen et al. (37)	Sichuan	J	340	176/164	N/A	convenience sampling	Journal	SSRS	N/A	N/A	HKRA
Wu et al. (38)	Guangdong	P, J	427	N/A	11–16	random	Journal	SSRS	N/A	SES	N/A
Wang (39)	Gansu	P	94	52/42	N/A	random	Dissertation	SSRS	CLS	N/A	N/A
Li and Guo (40)	Anhui	P	298	215/83	N/A	stratified sampling	Journal	SSRS	MHT	N/A	N/A
Zhou et al. (41)	Guangxi	J	523	186/337	14.24 ± 1.13	cluster sampling	Journal	SSRS	N/A	N/A	HKRA
Chen et al. (42)	Zhejiang	P, J, S	355	206/149	N/A	convenience sampling	Journal	SSRS	MHT	N/A	N/A
Zhao et al. (43)	Henan	P, J	218	N/A	11–16	convenience sampling	Journal	SSQ	N/A	SES	N/A
Ai and Hu (14)	Hunan, Sichuan	P	414	214/200	10.9 ± 1.07	convenience sampling	Journal	PSSS	CLS	N/A	CD-RISC
Niu (21)	Henan	P	356	220/136	N/A	convenience sampling	Dissertation	PSSS	N/A	N/A	HKRA
Chen and Zhao (44)	Zhejiang	P	335	170/185	N/A	convenience sampling	Journal	SSRS	N/A	SES	N/A
Yue and Lu (45)	Jiangsu, Guizhou	P	387	N/A	10–16	cluster, random sampling	Journal	SSRS	CLS	N/A	N/A
Zhao (46)	Guizhou	N/A	366	192/174	13.23 ± 1.13	random sampling	Journal	SSRS	CLS	N/A	N/A
Xiao and Zhang (47)	Jiangxi	N/A	437	214/223		convenience sampling	Journal	SSRS	CLS	N/A	N/A
Xiao (48)	Guangxi	P, J	1,110	473/637	N/A	cluster sampling	Dissertation	SSRS	N/A	N/A	ERS
Kong et al. (16)	Shandong	J	474	206/268	N/A	cluster, random sampling	Journal	SSRS	UCL-8	N/A	N/A
Lin and Bai (20)	Fujian	J	102	50/52	N/A	random	Journal	SSRS	N/A	N/A	CD-RISC
Ma and Yu (49)	Guizhou	J	763	347/415	16.12 ± 1.52	convenience sampling	Journal	PSSS	N/A	SES	RSCA
Fu (50)	Yunnan	J	182	94/88	14.3	cluster sampling	Journal	SSRS	N/A	SES	N/A
Ji et al. (51)	Sichuan	J	1,596	702/884	15.77 ± 0.74	convenience sampling	Journal	SSRS	N/A	SES	N/A
Liu (52)	Hubei	J	280	140/140	N/A	cluster sampling	Journal	SSRS	MHT	N/A	N/A
Man et al. (53)	Hunan	J	1,309	661/648	14.44 ± 1.14	cluster, random sampling	Journal	PSSS	N/A	SES	N/A
Qiao (54)	Yunnan	S	285	125/160	N/A	convenience sampling	Dissertation	PSSS	N/A	N/A	HKRA
Liu and Chen (55)	Guizhou	P	301	N/A	N/A	Random	Journal	SSRS	N/A	CSES	N/A
Shen (56)	Hubei	P	343	196/147	N/A	convenience sampling	Dissertation	SSRS	CLS	N/A	N/A
Fan and Lu (57)	Anhui	P, J	476	244/232	12 ± 1.80	Random	Journal	MSPSS	N/A	N/A	CYRM-28
Yu and Xiang (58)	Sichuan	J	377	N/A	N/A	convenience sampling	Journal	SSRS	N/A	N/A	RSCA
Fang (59)	Guangxi	J	1045	535/510	N/A	convenience sampling	Dissertation	SSRS	MHT	N/A	CD-RISC
Cheng et al. (15)	Anhui	J	220	114/106	13.67 ± 1.04	Random	Journal	SSRS	N/A	SES	N/A
Fan (60)	Guizhou	P	191	N/A	N/A	Random	Dissertation	SSRS	N/A	CSES	N/A
Hua et al. (61)	Eight provinces in central China	N/A	2,188	1,062/1,126	N/A	convenience sampling	Journal	SSRS	CLS	N/A	N/A
Li et al. (62)	Hunan	P, J	797	401/396	12.0 ± 2.0	Random	Journal	PSSS	UCL-8	N/A	N/A
Yang (63)	Yunnan	P, J, S	252	145/107	N/A	convenience sampling	Dissertation	PSSS	N/A	N/A	RSCA
Wang (64)	Henan	P	204	N/A	N/A	Random	Dissertation	SSQ	N/A	N/A	ERS
Ge and Liu (65)	Guizhou	J	316	N/A	N/A	cluster sampling	Journal	SSQ	N/A	N/A	RSCA
Ma and Gao (19)	Guizhou	J	980	461/519	16.12 ± 1.52	convenience sampling	Journal	PSSS	N/A	SES	RSCA
Huang et al. (66)	Eight cities/counties in central China	N/A	307	147/160	N/A	stratified random	Journal	SSRS	N/A	SES	N/A
Fan and Fan (67)	Hunan	P, J	692	326/366	11.99 ± 1.73	snowball sampling scheme	Journal	MSPSS	ALS	SES	ERS
Ma et al. (67)	Guizhou	J	1,095	528/567	16.46 ± 1.76	convenience sampling	Journal	PSSS	N/A	SES	RSCA

*P, Primary school; J, junior high school; S, Senior high school. SSRS, Social Support Rating Scale; SSQ, Social Support Questionnaire; MSPSS, Multi-dimensional Scale of Perceived Social Support; PSSS, Perceived Social Support Scale; CLS, Children Loneliness Scale; ULS-8, University of California Los Angeles Loneliness Scale-8; MHT, Mental Health Diagnostic Test; SES, Self-Esteem Scale; ALS, Adolescents' loneliness scale; CSES, Collective Self-Esteem Scale; ERS, Ego-Resiliency Scale; HKRA, Health Kids Resilience Assessment; CD-RISC, Connor-Davidson resilience scale; RSCA, Resilience Scale for Chinese Adolescents; CYRM-28, the Child and Youth Resilience Measure-28*.

**Figure 2:**
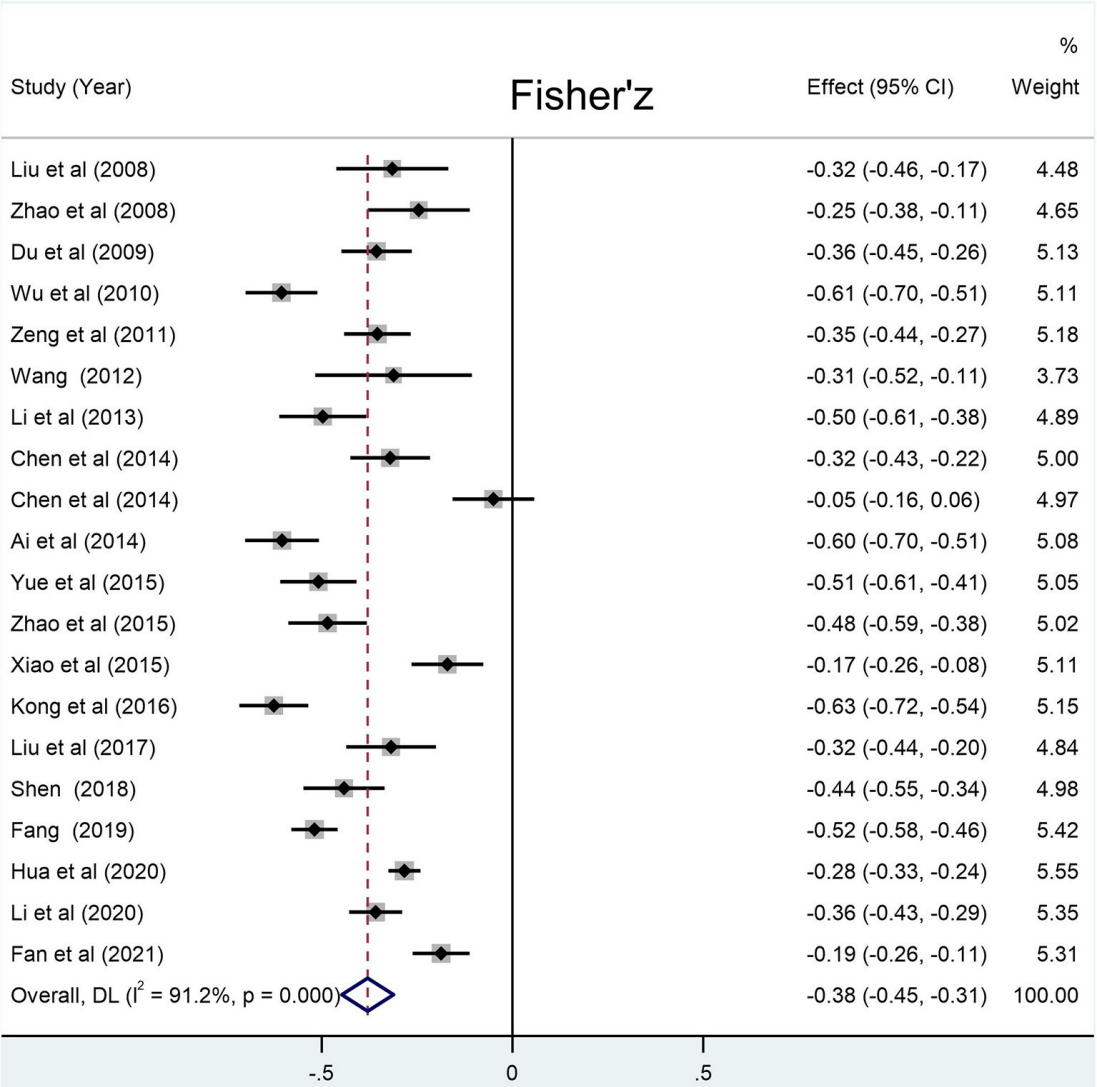
Forest plots for the correlation between social support and loneliness. Weights are from random-effects model.

**Figure 3:**
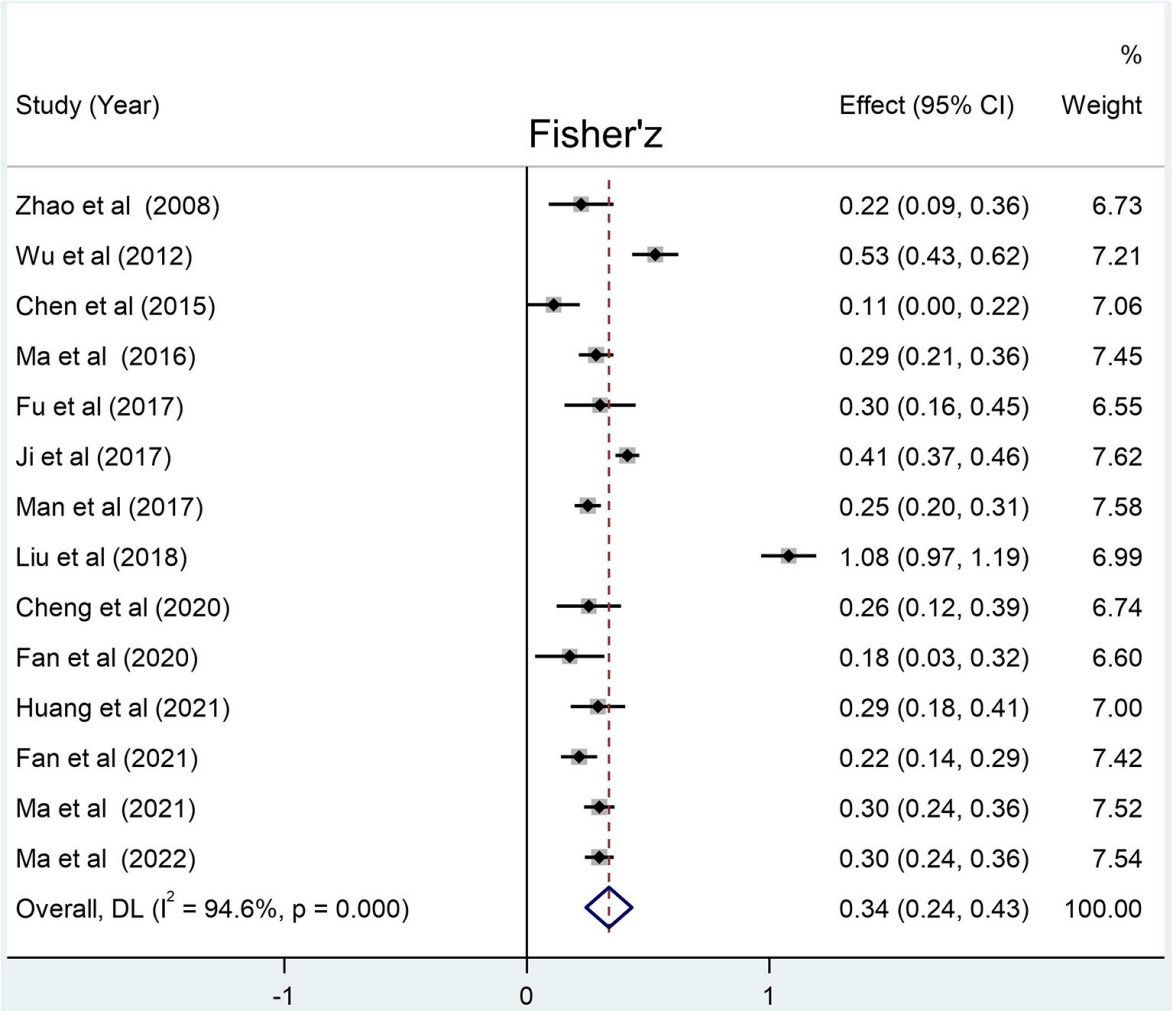
Forest plots for the correlation between social support and self-esteem. Weights are from random-effects model.

**Figure 4:**
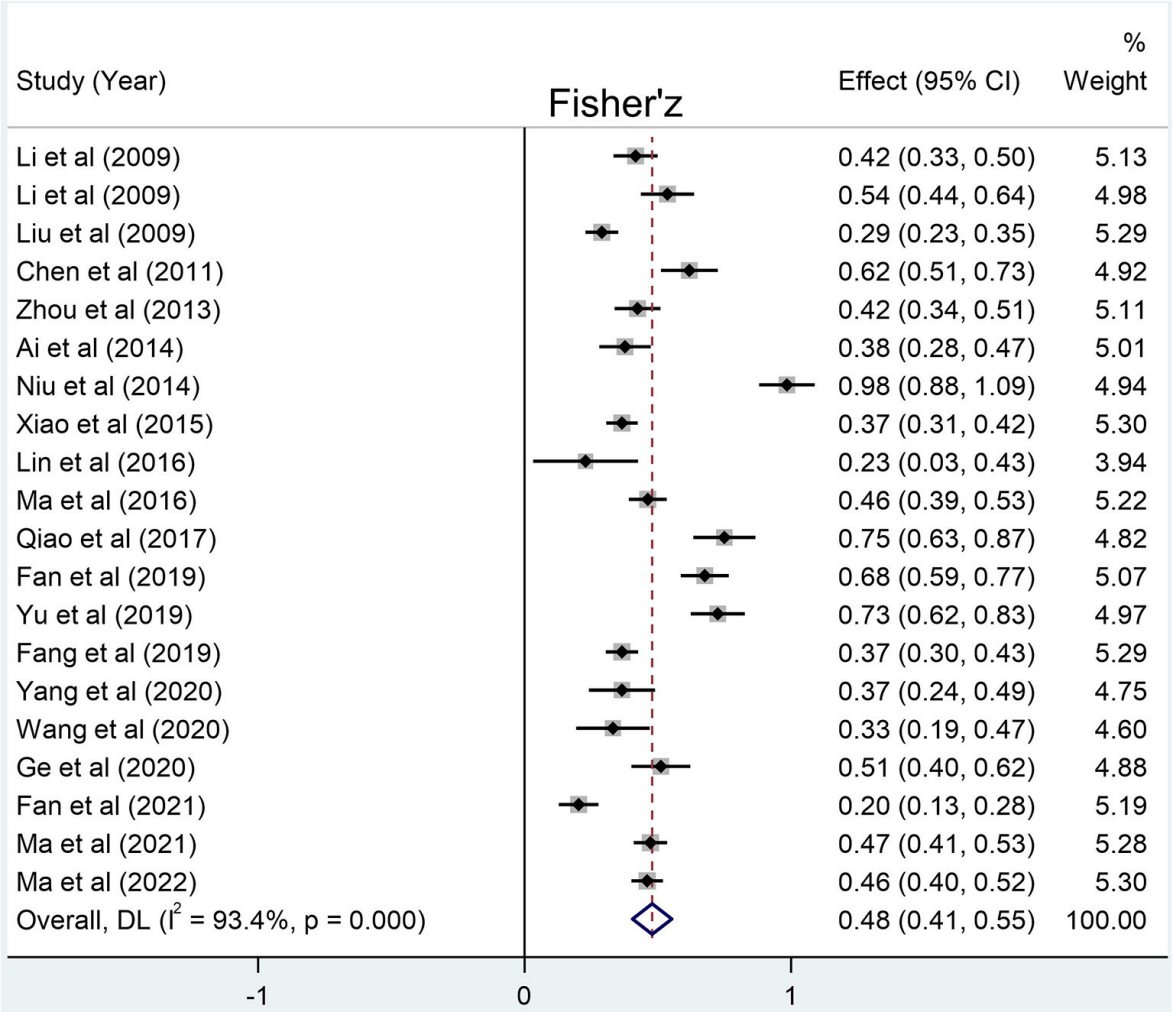
Forest plots for the correlation between social support and resilience. Weights are from random-effects model.

The number of LBC involved in the correlations between social support and loneliness, self-esteem, and resilience was 10 305, 8 615, and 11 292, respectively. The results of meta-analysis show that the social support of the LBC in mainland China has a relatively large negative correlation with loneliness (summary r: −0.36 [95% CI: −0.42– −0.30], p < 0.001), and a relatively large positive correlation with self-esteem [summary r: 0.33 (95% CI: 0.24– −0.41), *p* < 0.001] and resilience [summary r: 0.45 (95% CI: 0.38– −0.50), *p* < 0.001] (Figures 2–4).

### Subgroup Analysis

As shown in Table 2, the summary correlation coefficient between social support and loneliness did not reveal any significant difference when stratified by sample size, gender, education, published type, and social support measurement instrument (all with *p* > 0.05). However, we found that the summary correlation coefficient for loneliness in studies using random sample were higher than that in studies using non-random sample (random: summary r = −0.44, 95% CI: −0.51– −0.36, p < 0.001; non-random: summary r = −0.32, 95% CI: −0.39– −0.24, *p* < 0.001; Q_B_ = 4.81, *p* < 0.05). Similarly, studies using the ULS-8 measurement tool yielded higher negative correlation than studies using the CLS, MHT, and ALS measurement tools (ULS-8: summary r = −0.45, 95% CI: −0.64– −0.22, *p* < 0.001; CLS: summary r = −0.38, 95% CI: −0.46– −0.29, *p* < 0.001; MHT: summary r = −0.33, 95% CI: −0.43–−0.23, *p* < 0.001; ALS: summary r = −0.19, 95% CI: −0.26– −0.11, *p* < 0.001; Q_B_ = 15.08, *p* < 0.05).

**Table 2:** Subgroup analyses of the summary correlation between social support and loneliness among LBC.

	**Q_**BET**_**	**k**	***N***	**Summary r**	**95% CI**	**Q_**W**_**	**I^**2**^**
**Random sampling**	4.81*						
Yes		7	3,010	−0.44	[−0.51, −0.36]	36***	84.2%
No		13	7,295	−0.32	[−0.39, −0.24]	138.61***	91.3%
**Sample size**	0.58						
≤ 400		10	2,860	−0.33	[−0.42, −0.25]	58.55***	84.6%
>400		10	7,445	−0.38	[−0.46, −0.30]	157.23***	94.3%
**Gender**	3.74						
Male predominance (>50%)		8	3,712	−0.42	[−0.48, −0.36]	29.91***	76.6%
Female predominance (>50%)		7	4,933	−0.32	[−0.41, −0.22]	69.22***	91.3%
**Education**	5.16						
Primary school		5	1,536	−0.46	[−0.52, −0.39]	10.83**	54.7%
>Primary school		6	3,098	−0.36	[−0.48, −0.22]	82.80***	94.0%
Unclassified		6	2,680	−0.32	[−0.43, −0.21]	49.11***	89.8%
**Published type**	2.04						
Journal		17	8,823	−0.35	[−0.41, −0.29]	188.56***	91.5%
Dissertation		3	1,482	−0.42	[−0.50, −0.34]	24.57**	56.2%
**Social support measurement instrument**	3.23						
SSQ		2	399	−0.27	[−0.36, −0.18]	0.47	0.0%
SSRS		15	8,003	−0.37	[−0.44, −0.30]	165.51***	91.5%
MSPSS or PSSS		3	1,903	−0.36	[−0.54, −0.16]	44.88***	95.5%
**Loneliness measurement instrument**	15.08*						
CLS		10	5,065	−0.38	[−0.46, −0.29]	98.35 ***	90.8%
MHT		7	3277	−0.33	[−0.43, −0.23]	63.56***	90.6%
ULS-8		2	1,271	−0.45	[−0.64 −0.22]	21.07***	95.3%
ALS		1	692	−0.19	[−0.26 −0.11]	0.00	0.0%

Q_BET_: Between groups Q; Q_W_: Within groups Q; ^*^P < 0.05; ^**^P < 0.01; ^***^P < 0.001.

As shown in Table 3, subgroup analysis showed that sampling method, sample size, gender, education, publication type, social support measurement tool, and self-esteem measurement tool did not significantly affect the relationship between social support and self-esteem (all with *p* > 0.05).

**Table 3:** Subgroup analyses of the summary correlation between social support and self-esteem among LBC.

	**Q_**BET**_**	**k**	***N***	**Summary r**	**95% CI**	**Q_**W**_**	**I^**2**^**
**Random sampling**	1.40						
Yes		6	2,755	0.40	[0.18, 0.59]	190.33***	97.4%
No		8	5,860	0.27	[0.21, 0.33]	38.89*	82.0%
**Sample size**	0.03						
≤ 400		7	1,754	0.34	[0.08, 0.55]	189.45***	96.8%
>400		7	6,861	0.31	[0.25, 0.37]	48.33***	87.6%
**Gender**	0.29						
Male predominance (>50%)		3	1,711	0.25	[0.21, 0.29]	0.41***	0.0%
Female predominance (>50%)		7	5,767	0.27	[0.21, 0.33]	37.47**	84.0%
**Education**	0.24						
Primary school		3	827	0.43	[0.18, 0.69]	169.71**	98.8%
>Primary school		7	6,144	0.30	[0.25, 0.34]	23.01***	73.9%
Unclassified		3	1,337	0.31	[0.11, 0.49]	28.15***	92.9%
**Published type**	3.82						
Journal		13	8,424	0.34	[0.25, 0.42]	236.35***	94.9%
Dissertation		1	191	0.18	[0.03, 0.31]	0.00	0.0%
**Social support measurement instrument**	3.23						
SSQ		1	218	0.22	[0.09, 0.36]	0.00	0.00
SSRS		8	3,559	0.37	[0.21, 0.53]	190.91***	91.5%
MSPSS or PSSS		5	4,838	0.27	[0.24, 0.29]	4.38	95.5%
**Self-esteem measurement instrument**	0.53						
SES		12	8,123	0.28	[0.23, 0.34]	58.20***	84.5%
CSES		2	492	0.55	[−0.25, 0.90]	93.92***	98.9%

Q_BET_: Between groups Q; Q_W_: Within groups Q; ^*^P < 0.05; ^**^P < 0.01; ^***^P < 0.001.

The summary correlation coefficient between social support and resilience was substantially changed when stratified by the sample size and measurement instrument for resilience (all with *p* < 0.05). No difference was observed in subgroup analyses by sampling strategy, gender, education, published type or instrument for social support (all with *p* > 0.05) (Table 4).

**Table 4:** Subgroup analyses of the summary correlation between social support and resilience among LBC.

	**Q_**BET**_**	**k**	***N***	**Summary r**	**95% CI**	**Q_**W**_**	**I^**2**^**
**Random sampling**	0.07						
Yes		4	1,168	0.43	[0.27, 0.56]	26.99***	88.9%
No		16	10,124	0.45	[0.38, 0.51]	253.27***	94.1%
**Sample size**	3.90*						
≤ 400		9	2,618	0.51	[0.40, 0.61]	109.96***	92.7%
>400		11	8,674	0.39	[0.33, 0.44]	90.36***	88.9%
**Gender**	2.48						
Male predominance (>50%)		8	3,830	0.49	[0.38, 0.59]	135.88***	94.8%
Female predominance (>50%)		9	6,565	0.39	[0.31, 0.46]	90.06***	91.1%
**Education**	0.80						
Primary school		3	974	0.51	[0.14, 0.76]	87.02**	97.7%
>Primary school		13	7,788	0.45	[0.39, 0.50]	107.01***	88.8%
Unclassified		4	2,530	0.38	[0.21, 0.53]	63.42***	95.3%
**Published type**	0.04						
Journal		13	7,024	0.44	[0.38, 0.50]	115.29***	89.6%
Dissertation		7	4,268	0.46	[0.31, 0.58]	168.98***	96.4%
**Social support measurement instrument**	0.49						
SSQ		2	520	0.40	[0.25, 0.54]	3.95*	74.7%
SSRS		8	4,444	0.43	[0.36, 0.51]	61.44***	88.6%
MSPSS or PSSS		10	6,328	0.46	[0.36, 0.56]	222.92***	96.0%
**Resilience measurement instrument**	46.54***						
ERS		6	3,969	0.34	[0.26, 0.41]	34.25***	85.4%
HKRA		4	1,504	0.60	[0.42, 0.73]	69.06***	95.7%
CD-RISC		3	1,561	0.35	[0.30, 0.39]	1.85	0.0%
RSCA		6	3,782	0.46	[0.40, 0.52]	26.52***	81.1%
CYRM-28		1	476	0.59	[0.53 0.64]	0.00	0.0%

Q_BET_: Between groups Q; Q_W_: Within groups Q; ^*^P <0.05; ^**^P <0.01; ^***^P <0.001.

### Sensitivity Analyses

Stability of results was assessed by sequentially excluding one study and then recalculating the pooled correlation coefficient. Sensitivity analyses for summary correlation coefficients between social support and loneliness, self-esteem, and resilience revealed minor changes, indicating that our results were stable (Supplementary File 3).

### Publication Bias

Judging subjectively, it was difficult to determine whether the funnel plots for the summary correlation coefficients between social support and loneliness, self-esteem, and relisience were symmetric or not (Supplementary File 4). The Egger linear regression test showed insignificant results (Loneliness: t = −0.51, *p* = 0.62; Self-esteem:t = 0.23, *p* = 0.82;Resilience:t = 1.62, *p* = 0.12). Duval and Tweedle trim-and-fill procedure suggested that no additional research is needed for all three meta-analyses (Supplementary File 5). Taken together, it suggests the absence of publication bias for these meta-analyses.

## Discussion

To the best of our knowledge, this was the first meta-analysis exploring the pooled correlation coefficients of social support with loneliness, self-esteem, and resilience among LBC in mainland China. Our results indicated that the social support of the LBC in mainland China has a relatively large negative correlation with loneliness, and a relatively large positive correlation with self-esteem and resilience, with a series of summary Pearson's correlation coefficients of −0.36, 0.33 and 0.45, respectively. The sensitivity analysis results are robust, which indicates that the pooled analysis of correlation coefficients is reliable and convincing. Egger linear regression and Duval and Tweedie's trim-and-fill analysis also show that there is no publication bias in our research results.

According to interactionist theory, loneliness is a response to the lack of satisfactory social networks (68). Social support can fill the gap between social network and social contact needs, thus reducing loneliness (69). Research by Ayalon, Shiovitz-Ezra and Palgi suggests that the frequency, content and meaning of social interactions have a significant impact on loneliness (70). The effect of social support on loneliness is also likely to be mediated by other variables, not just directly. Evidence has shown that the association between social support and loneliness could be mediated by gratitude (71). According to the broaden-and-build theory of positive emotions, gratitude as a positive emotion helps individuals to expand their cognitive schema, enhance the flexibility of activities, construct personal psychological and social resources, and eliminate the negative effects of negative emotions (72). Individuals with higher social support levels have more positive emotional experiences such as gratitude, which reduces their loneliness. Interestingly, there is also evidence that psychopathology in turn affects social support (73). The social selection model believes that healthy individuals are more likely to obtain good social relationships, experience more social support, and have more positive evaluations of themselves, others, and the world (74), which may increase individuals' gratitude to others and stimulate others' willingness and behavior to further support individuals (75), thus enabling individuals to experience more social support. Adolescence is a transitional stage from children to adults, when individuals face more psychological conflicts and pressures (32). On the one hand, the psychological sense of adulthood caused by teenagers' physiological maturity coexists with naivety in reality, which makes it easy for teenagers to experience psychological conflicts; On the other hand, adolescent individuals begin to assume some adult roles and obligations (32). This change of social role makes them pay attention to the practical problems of future development, such as further education and employment, and bear great pressure from family, school and society. For LBC who are in the background of relative lack of parental affection, the psychological conflicts and pressures they experience may be more serious than those of ordinary teenagers, so they are more likely to have psychological problems (76). LBC is more prone than NLBC to experience psychological difficulties such as loneliness, according to studies (76). Our findings suggest that there is a substantial negative relationship between social support and loneliness among LBC, and that enhancing social support for these children can help them feel less lonely. As a result, establishing a proper social support system for the mental health development of the LBC is critical.

Consistent with previous literature, self-esteem was positively correlated with social support. Adela's research found that social support is beneficial to improve an individual's self-esteem (77). Rosenberg also put forward a similar point of view, he believed that self-esteem is the result of social support, in adolescence, a harmonious social environment has a very important role in the maintenance of self-esteem (78). Yarcheski's study of 165 adolescents aged 15–17 found that the correlation between social support and self-esteem was 0.30 (79); in 1997, they reported a correlation of 0.38 (80). For LBC in an unfavorable growing environment, they may not be able to receive timely support and affirmation from their parents like NLBC. This kind of support is not satisfied for a long time, which will lead to the LBC often denying themselves, having a low evaluation of self-worth and developing a low self-esteem personality (44). Studies have shown that compared with NLBC, LBC show lower self-esteem (81). The positive correlation between social support and self-esteem reveals that for the LBC who lack parental care, increasing social support may be an effective strategy to help LBC enhance their positive self-identity and improve their self-esteem.

The results of the meta-analysis show that social support is positively correlated with resilience, which is consistent with the results of previous study (82). The resilience is the psychological function that an individual can keep or return to normal after experiencing adversity or trauma (83). It is a successful “self-adjustment mechanism” response and an important protective element against emotional and behavioral issues in adolescents (84). The main effect model of social support believes that social support has a general positive effect, no matter what the current level of social support is, as long as social support increases, it will inevitably lead to the improvement of individual health status (85). In addition, the positive correlation between the two is also consistent with the prediction of the protective-protective model, that one protective factor enhances the effect of another protective factor (86). Research shows that the ability of LBC to adapt to the difficulties such as lack of parental affection, insufficient family education and limitation of intergenerational rearing is related to psychological resilience (87). This suggests that we should provide a good social support environment for the children who have been left behind. LBC can gain problem-solving skills and experience from family, friends, schools and other outsiders by establishing a safe and friendly relationship with others, which will have a positive effect on their psychological resilience, thus effectively buffering the influence of external harmful factors.

Moderating effect analysis showed that sampling methods and loneliness measurement tools may moderate the relationship between social support and loneliness. The former suggests that the quality of research design may affect the relationship between the two, and more accurate conclusions may be obtained by using random sampling methods. The latter may be due to the lack of included literature in some subgroups (e.g., the ULS-8 and ALS groups included only 2 and 1 articles, respectively). Subgroup analysis by sample size showed that the summary social support-resilience correlation coefficients were higher in smaller sample size studies than in larger sample size studies (summary r: 0.51 vs. 0.39, *p* <0.01) (Table 3). Studies with larger sample sizes may be more representative and more likely to yield reliable results. Resilience measurement tools may also regulate the relationship between social support and resilience, which may also be related to the lack of literature in some groups (for example, CYRM-28 group only contains one literature).

## Limitations and Future Implications

When interpreting the results of the current study, several limitations must be kept in mind. First, to reduce the potential source of heterogeneity, social support measurement tool was restricted to the SSRS, SSQ, MSPSS, and PSSS. Similarly, measurement tool for loneliness was restricted to the CLS, ULS, MHT and ALS; the measurement instrument for self-esteem was restricted to the SES and CSES. As a result, the article included in this meta-analysis was limited. In the future, after the paper is further enriched, it is necessary to further explore the research using other measurement tools. Second, in our search strategy, only a few commonly used databases were searched, which may lead to certain publication bias. Future research can increase the number of retrieval databases, such as Scopus, EBSCO, Springer link, The Cochrane library, to minimize the risk of bias in research results. Third, in view of the limited number of studies included, subgroup analysis based on some moderators should be carefully interpreted to some extent. In addition, even after subgroup analysis, the aggregate correlation coefficient still has substantial heterogeneity. Other possible influencing factors such as pre-existing illness, personality, comorbidity, lifestyle, and living conditions might also account for this correlation. Unfortunately, because the effect size is Pearson correlation coefficient rather than partial correlation coefficient, the correlation between social support and the three clinical variables is calculated without adjusting the related variables. Few studies conducted stratified Pearson's correlation analysis according to these variables. Future research can further focus on other variables (such as personality, living conditions.) that may directly affect social support and loneliness, self-esteem, and resilience in Chinese LBC, so as to provide clearer intervention ideas for future mental health research. Finally, the articles included in this study are all cross-sectional studies, and no judgment can be made on the causal relationship between variables. Therefore, more longitudinal studies are still needed in the future to test the causal relationship between social support and loneliness, self-esteem, and resilience.

## Conclusion

Despite the limitations mentioned above, all available evidence supports that the social support of the LBC in mainland China has a relatively large negative correlation with loneliness, and a relatively large positive correlation with self-esteem and resilience. Their summary Pearson's correlation coefficients were −0.36, 0.33 and 0.45, respectively. This means that left-behind children in mainland China with high levels of social support are more likely to have lower levels of loneliness and higher levels of self-esteem and resilience. More studies, especially large prospective studies with long follow-up periods, are warranted to verify our findings.

## Data Availability Statement

The data analyzed in this study is subject to the following licenses/restrictions: The datasets presented in this study are available from the authors upon reasonable request. Requests to access these datasets should be directed to HH, huangtaomiss@163.com.

## Author Contributions

HH and XW: study design, critical revision of the manuscript, and drafting of the manuscript. YL, YZ, QP, YD, GL, and CC: analysis and interpretation of data. All authors approval of the final version for submission.

## Funding

This research was sponsored by Graduate Education Reform and Quality Improvement Project of Henan Province (Grant Number: YJS2021AL074), Graduate Education Innovation and Quality Improvement Project of Henan University (Grant Number: SYL19060141), and Planning and Decision Consultation Project of Henan Province (Grant Number: 2018JC38).

## Conflict of Interest

The authors declare that the research was conducted in the absence of any commercial or financial relationships that could be construed as a potential conflict of interest.

## Publisher's Note

All claims expressed in this article are solely those of the authors and do not necessarily represent those of their affiliated organizations, or those of the publisher, the editors and the reviewers. Any product that may be evaluated in this article, or claim that may be made by its manufacturer, is not guaranteed or endorsed by the publisher.

## Supplementary Material

Supplementary File 1
Search strategy.

Supplementary File 2
Quality assessment form for included studies.

Supplementary File 3
Sensitivity analyses of correlations between social support and loneliness, self-esteem, and resilience.

Supplementary File 4
Funnel plot of correlations between social support and loneliness, self-esteem, and resilience.

Supplementary File 5
Duval and Tweedle trim-and-fill procedure of correlations between social support and loneliness, self-esteem, and resilience.

Supplementary File 6
PRISMA Checklist.
