# Supplementary material for: Correlations Between Social Support and Loneliness, Self-Esteem, and Resilience Among Left-Behind Children in Mainland China: A Meta-Analysis
Source: Front Psychiatry. 2022 Apr 27;13:874905. doi: 10.3389/fpsyt.2022.874905 (PMC9095419; doi:10.3389/fpsyt.2022.874905)
Supplement: Supplementary File 1 — Search strategy. [file Data_Sheet_1.docx]

**SEARCH STRATEGY IN PUBMED DATABASE**

| **Search** | **Query** | **Results** | **Time** |
| --- | --- | --- | --- |
| **#1** | **(left-behind[Title/Abstract]) OR (stay at home[Title/Abstract])** | **4546** | **05:58:54** |
| **#2** | **"child*"[Title/Abstract] OR "adolescent*"[Title/Abstract] OR "student*"[Title/Abstract]** | **1,981,036** | **05:59:16** |
| **#3** | **"support"[Title/Abstract] OR "social support"[Title/Abstract] OR "social network"[Title/Abstract] OR "social relation"[Title/Abstract] OR "social resource"[Title/Abstract] OR "social environment"[Title/Abstract]** | **1,136,943** | **05:59:32** |
| **#4** | **"China"[Title/Abstract] OR "Chinese"[Title/Abstract]** | **424,775** | **05:59:46** |
| **#5** | **("left-behind"[Title/Abstract] OR "stay at home"[Title/Abstract]) AND ("child*"[Title/Abstract] OR "adolescent*"[Title/Abstract] OR "student*"[Title/Abstract]) AND ("support"[Title/Abstract] OR "social support"[Title/Abstract] OR "social network"[Title/Abstract] OR "social relation"[Title/Abstract] OR "social resource"[Title/Abstract] OR "social environment"[Title/Abstract]) AND ("China"[Title/Abstract] OR "Chinese"[Title/Abstract])** | **72** | **06:00:19** |

**SEARCH STRATEGY IN EMBASE DATABASE**

| **History** | **Query** | **Results** |
| --- | --- | --- |
| **#1** | **'left-behind':ab,ti** | **3,312** |
| **#2** | **'stay at home':ab,ti** | **1,862** |
| **#3** | **'child*':ab,ti** | **1,989,499** |
| **#4** | **'adolescent*':ab,ti** | **372,379** |
| **#5** | **'student*':ab,ti** | **420,181** |
| **#6** | **'social support':ab,ti** | **54,805** |
| **#7** | **'social network':ab,ti** | **12,080** |
| **#8** | **'social relation':ab,ti** | **158** |
| **#9** | **'social resource':ab,ti** | **139** |
| **#10** | **'social environment':ab,ti** | **88,06** |
| **#11** | **'chinese':ab,ti** | **312,456** |
| **#12** | **'china':ab,ti** | **266,195** |
| **#13** | **#1 OR #2** | **5,173** |
| **#14** | **#3 OR #4 OR #5** | **2,543,718** |
| **#15** | **#6 OR #7 OR #8 OR #9 OR #10** | **73,527** |
| **#16** | **#11 OR #12** | **511,817** |
| **#17** | **#13 AND #14 AND #15 AND #16** | **22** |
