# Supplementary material for: Correlations Between Social Support and Loneliness, Self-Esteem, and Resilience Among Left-Behind Children in Mainland China: A Meta-Analysis
Source: Front Psychiatry. 2022 Apr 27;13:874905. doi: 10.3389/fpsyt.2022.874905 (PMC9095419; doi:10.3389/fpsyt.2022.874905)
Supplement: Supplementary File 2 — Quality assessment form for included studies. [file Data_Sheet_2.docx]

**JBI CRITICAL APPRAISAL CHECKLIST FOR STUDIES REPORTING PREVALENCE DATA**

| Items | Yes | No | Unclear | Not applicable |
| --- | --- | --- | --- | --- |
| 1.Was the sample frame appropriate to address the target population? |  |  |  |  |
| 2.Were study participants sampled in an appropriate way? |  |  |  |  |
| 3.Was the sample size adequate? |  |  |  |  |
| 4.Were the study subjects and the setting described in detail? |  |  |  |  |
| 5.Was the data analysis conducted with sufficient coverage of the identified sample? |  |  |  |  |
| 6.Were valid methods used for the identification of the condition? |  |  |  |  |
| 7.Was the condition measured in a standard, reliable way for all participants? |  |  |  |  |
| 8.Was there appropriate statistical analysis? |  |  |  |  |
| 9.Was the response rate adequate, and if not, was the low response rate managed appropriately? |  |  |  |  |

Quality assessment adapted from: Munn Z, Moola S, Lisy K, Riitano D, Tufanaru C. Methodological guidance for systematic reviews of observational epidemiological studies reporting prevalence and incidence data. Int J Evid Based Healthc. 2015;13(3):147–153.

**QUALITY ASSESSMENT FOR THE 47 STUDIES IN THE CURRENT META-ANALYSIS.**

| Study(year) | Quality Item | | | | | | | | | |
| --- | --- | --- | --- | --- | --- | --- | --- | --- | --- | --- |
|  | ① | ② | ③ | ④ | ⑤ | ⑥ | ⑦ | ⑧ | ⑨ | Total |
| Liu et al 2008 | Y | N | Y | Y | Y | N | Y | Y | Y | 7 |
| Zhao et al 2008 | Y | U | Y | N | Y | Y | Y | Y | Y | 7 |
| Chen et al 2014 | Y | N | Y | N | Y | Y | N | Y | Y | 6 |
| Du et al 2009 | Y | N | Y | N | Y | N | Y | Y | Y | 6 |
| Li et al 2009 | Y | Y | Y | Y | Y | N | Y | Y | Y | 8 |
| Li et al 2009 | Y | Y | Y | N | Y | N | Y | Y | Y | 7 |
| Liu 2009 | Y | N | Y | Y | Y | N | U | Y | Y | 6 |
| Wu et al 2010 | Y | Y | Y | Y | Y | Y | N | Y | Y | 8 |
| Zeng et al 2011 | Y | U | Y | Y | Y | N | Y | Y | N | 6 |
| Chen et al 2011 | Y | Y | Y | N | Y | N | Y | Y | Y | 7 |
| Wu et al 2012 | Y | Y | Y | N | Y | Y | N | Y | Y | 7 |
| Wang 2012 | Y | N | Y | N | Y | N | Y | Y | Y | 6 |
| Li et al 2013 | Y | Y | Y | Y | Y | N | Y | Y | Y | 8 |
| Zhou et al 2013 | Y | Y | Y | Y | Y | Y | Y | Y | Y | 9 |
| Chen et al 2014 | Y | Y | Y | Y | Y | Y | N | Y | Y | 8 |
| Zhao et al 2008 | Y | N | Y | Y | Y | Y | N | Y | Y | 7 |
| Ai et al 2014 | Y | Y | Y | Y | Y | Y | Y | Y | Y | 9 |
| Niu et al 2014 | Y | Y | Y | N | Y | N | Y | Y | Y | 7 |
| Chen et al 2015 | Y | N | Y | Y | Y | N | U | Y | Y | 6 |
| Yue et al 2015 | Y | Y | Y | Y | Y | Y | N | Y | Y | 8 |
| Zhao et al 2015 | Y | U | Y | Y | Y | N | Y | Y | N | 6 |
| Xiao et al 2015 | Y | Y | Y | N | Y | N | Y | Y | Y | 7 |
| Xiao et al 2015 | Y | Y | Y | N | Y | Y | N | Y | Y | 7 |
| Kong et al 2016 | Y | U | Y | Y | Y | N | Y | Y | N | 6 |
| Lin et al 2016 | Y | Y | Y | N | Y | N | Y | Y | Y | 7 |
| Ma et al 2016 | Y | Y | Y | N | Y | Y | N | Y | Y | 7 |
| Fu et al 2017 | Y | N | Y | N | Y | N | Y | Y | Y | 6 |
| Ji et al 2017 | Y | U | Y | Y | Y | N | Y | Y | N | 6 |
| Liu et al 2017 | Y | Y | Y | N | Y | N | Y | Y | Y | 7 |
| Man et al 2017 | Y | Y | Y | Y | Y | Y | Y | Y | Y | 9 |
| Qiao et al 2017 | Y | N | Y | N | Y | N | Y | Y | Y | 6 |
| Liu et al 2018 | Y | Y | Y | Y | Y | N | Y | Y | Y | 8 |
| Shen et al 2018 | Y | Y | Y | Y | Y | Y | Y | Y | Y | 9 |
| Fan et al 2019 | Y | Y | Y | Y | Y | Y | N | Y | Y | 8 |
| Yu et al 2019 | Y | N | Y | Y | Y | Y | N | Y | Y | 7 |
| Fang et al 2019 | Y | Y | Y | Y | Y | Y | Y | Y | Y | 9 |
| Cheng et al 2020 | Y | Y | Y | N | Y | N | Y | Y | Y | 7 |
| Fan et al 2020 | Y | Y | Y | Y | Y | Y | Y | Y | Y | 9 |
| Hua et al 2020 | Y | N | Y | N | Y | N | Y | Y | Y | 6 |
| Li et al 2020 | Y | N | Y | N | Y | N | Y | Y | Y | 6 |
| Yang 2020 | Y | U | Y | Y | Y | N | Y | Y | N | 6 |
| Wang 2020 | Y | Y | Y | N | Y | N | Y | Y | Y | 7 |
| Ge et al 2020 | Y | Y | Y | Y | Y | Y | Y | Y | Y | 9 |
| Huang et al 2021 | Y | N | Y | N | Y | N | Y | Y | Y | 6 |
| Fan et al 2021 | Y | Y | Y | Y | Y | N | Y | Y | Y | 8 |
| Ma et al 2021 | Y | Y | Y | Y | Y | Y | Y | Y | Y | 9 |
| Ma et al 2022 | Y | Y | Y | Y | Y | Y | N | Y | Y | 8 |

**Abbreviations: Y, yes; N, No; U, unclear.**
