# Supplementary material for: Correlations Between Social Support and Loneliness, Self-Esteem, and Resilience Among Left-Behind Children in Mainland China: A Meta-Analysis
Source: Front Psychiatry. 2022 Apr 27;13:874905. doi: 10.3389/fpsyt.2022.874905 (PMC9095419; doi:10.3389/fpsyt.2022.874905)
Supplement: Supplementary File 4 — Funnel plot of correlations between social support and loneliness, self-esteem, and resilience. [file Data_Sheet_4.docx]

**Fig 1 Funnel diagram of the relationship between social support and loneliness**

**Fig 2 Funnel diagram of the relationship between social support and self-esteem**

**Fig 3 Funnel diagram of the relationship between social support and resilience**
